# Strain-Specific Reduction of Ndel1 Enzymatic Activity During Early Zika Virus Infection in the Neonatal Brain

**DOI:** 10.1007/s10571-026-01798-4

**Published:** 2026-08-17

**Authors:** Ingrid S. de Farias, Ana P. Ximenes, William Y. Oyadomari, Raissa Rilo Christoff, João V. S. Nani, Tailene Rabello, Luiza M. Higa, Patrícia P. Garcez, Mirian A. F. Hayashi

**Affiliations:** 1 Department of Pharmacology, Escola Paulista de Medicina (EPM), Universidade Federal de São Paulo (UNIFESP), R. Três de Maio, 100-Vila Clementino, São Paulo, SP 04044-020 Brazil; 2 Instituto de Ciências Biomédicas, Universidade Federal Do Rio de Janeiro (UFRJ), R. Manoel Frota Moreira, 1 andar—Cidade Universitária, Rio de Janeiro, RJ 21941-902 Brazil; 3 Department of Neuroimaging, School of Neuroscience, Institute of Psychiatry, Psychology & Neuroscience (IoPPN), King’s College London, 16 De Crespigny Park-Denmark Hill, London, SE5 8AF UK; 4 Instituto de Biologia - Laboratório de Virologia Molecular, Departamento de Genética, Universidade Federal Do Rio de Janeiro (UFRJ), Av. Carlos Chagas Filho, 373-Sala A1-050-Cidade Universitária, Rio de Janeiro, RJ 21941-902 Brazil

**Keywords:** Zika virus, Ndel1, Neuroinflammation, Congenital Zika syndrome, Neurodevelopment, Flavivirus infection

## Abstract

Zika virus (ZIKV) infection during early development is associated with severe neurodevelopmental impairment, including congenital microcephaly, yet the molecular mechanisms linking viral exposure to neuronal dysfunction remain incompletely understood. Ndel1 (nudE neurodevelopment protein 1 like 1) is an oligopeptidase critically involved in neuronal migration, neurite outgrowth, and cytoskeletal dynamics, processes essential for proper brain development. Building on previous observations that Ndel1 enzymatic activity is reduced in ZIKV-infected embryonic and neonatal brains, we investigated whether this modulation represents a conserved host response or a strain-specific molecular effect. Using a neonatal intracerebroventricular infection model in Swiss mice at postnatal day 0, we compared the effects of a Brazilian ZIKV strain (ZIKV^BR^), an African lineage strain (ZIKV^AF^), and dengue virus (DENV) on Ndel1 regulation in the brain. We found that Ndel1 enzymatic activity was significantly reduced at early post-infection time points (P3 and P7) exclusively in ZIKV^BR^-infected animals, whereas ZIKV^AF^ and DENV did not alter enzyme activity relative to controls. Notably, at the earliest timepoint (P3), this reduction occurred in the absence of changes in Ndel1 mRNA expression, indicating an initial post-transcriptional or functional mechanism of regulation, although Ndel1 mRNA expression was also significantly reduced by P7. Although both ZIKV strains induced robust inflammatory gene expression, only ZIKV^BR^ infection resulted in decreased Ndel1 activity, dissociating neuroinflammatory signaling from enzymatic modulation. Importantly, this strain-specific reduction parallels the unique capacity of ZIKV^BR^ to induce microcephaly and supports Ndel1 dysfunction as an early molecular event associated with neurodevelopmental impairment. Together, these findings identify a virus- and strain-specific regulation of a key neuronal enzyme and provide mechanistic insight into the molecular basis of ZIKV^BR^-associated neurodevelopmental outcomes.

**Graphical Abstract:**

**Figure:**
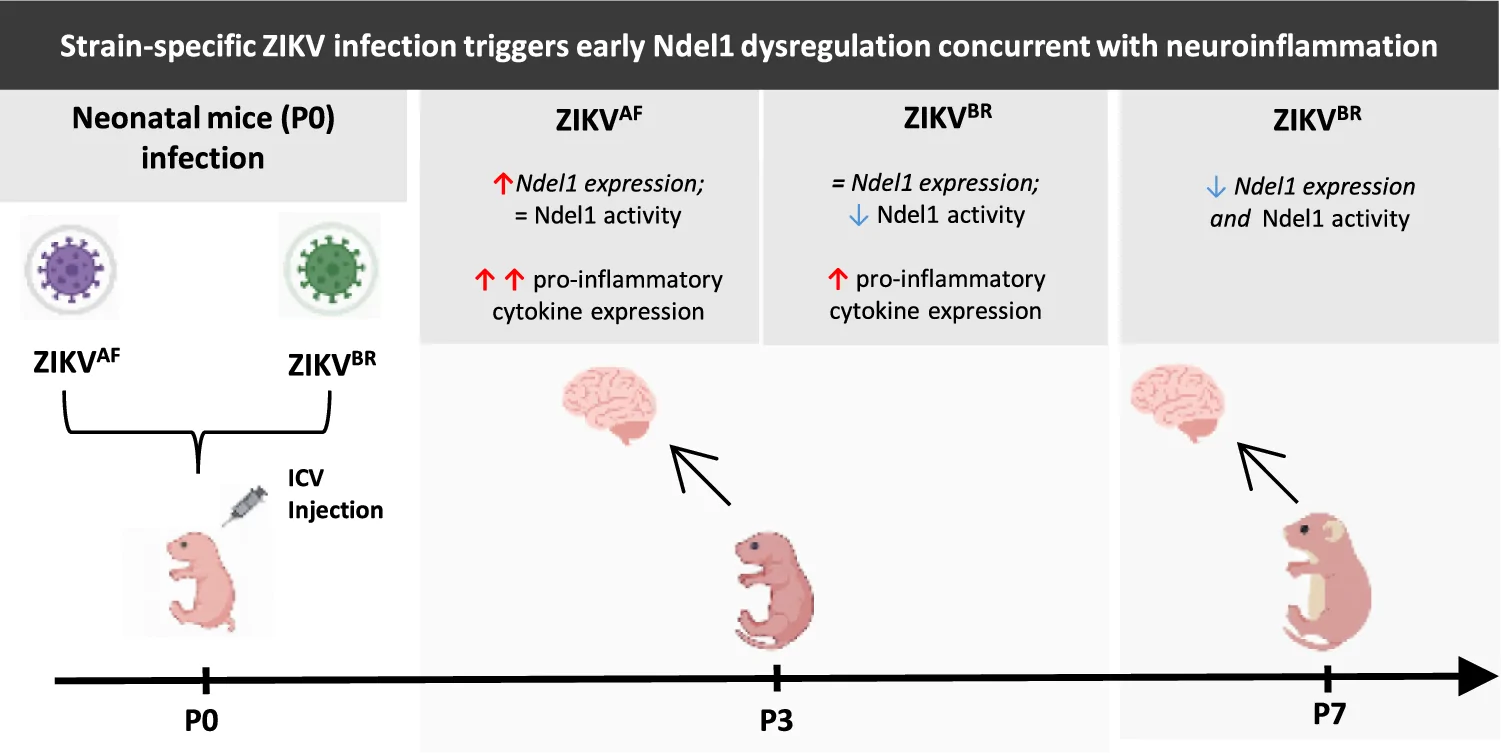

## Introduction

Flaviviruses have a long history of human infection, and their geographical spread has intensified in recent decades, increasingly impacting larger populations and posing a growing public health threat. The World Health Organization (WHO) has actively monitored over 1,400 epidemic events triggered by various emerging viruses across 172 countries in recent years, including influenza, severe acute respiratory syndrome (SARS), middle east respiratory syndrome (MERS), yellow fever, and Zika virus (ZIKV) (Vue and Tang 2021). The WHO has declared three global health emergencies in the last decade alone associated with emerging viruses, including Ebola virus (EBOV) in 2014, ZIKV in 2016, and SARS-CoV-2 in 2020 (Estévez-Herrera et al. 2021), underscoring the worldwide impact of viral outbreaks, which can rapidly halt economic production and claim numerous lives. Flavivirus infections, such as those caused by ZIKV and dengue virus (DENV), represent a significant global public health challenge due to their capacity to induce severe clinical manifestations and affect millions of individuals, especially in tropical countries including Brazil. This scenario is further exacerbated by the rapid geographic expansion of these arboviruses, driven by factors such as climate change, unplanned urbanization, and increased human mobility (Daudt-Lemos et al. 2025). Brazil records one of the highest incidences of DENV and ZIKV globally, reflecting a concerning pattern of co-circulation with overlapping outbreaks that significantly hinder effective control strategies.

Flaviviruses can interfere with brain formation and the spatial organization of neurons and synapses during neurodevelopment, potentially leading to severe neurological complications that may result in complex clinical manifestations such as cognitive and motor impairments, seizures, behavioral disorders, encephalopathies, and, ultimately, microcephaly (Cugola et al. 2016; Miner and Diamond 2017). In addition to their direct cytopathic effects, these viruses trigger robust innate immune responses within the central nervous system, leading to neuroinflammation that can further compromise neuronal survival, synaptic organization, and developmental processes. Although sharing similar vectors and initial symptoms, arboviruses can present specific and often severe complications. ZIKV is primarily associated with neurodevelopmental anomalies, such as congenital zika syndrome (CZS), particularly when maternal infection occurs during pregnancy (Filgueiras et al. 2021). In contrast, DENV infection can trigger severe hemorrhagic manifestations, including dengue hemorrhagic fever and dengue shock syndrome. Although less frequent but potentially lethal, neurological complications such as encephalitis and acute disseminated encephalomyelitis have also been reported, reflecting the capacity of these viruses to induce inflammatory responses in the central nervous system (Trivedi and Chakravarty 2022). The serological and symptomatic similarities between these co-circulating viruses, particularly ZIKV and DENV, further complicate differential diagnosis and epidemiological control, highlighting the urgent need to understand their diverse clinical impacts, neurotropic potential, and associated neuroimmune mechanisms (Culshaw et al. 2017).

A key unresolved question in this field concerns whether the neuropathogenic effects observed during flavivirus infections reflect shared mechanisms common to the family or virus- and strain-specific interactions with the host nervous system. This distinction is particularly relevant for ZIKV, given that different strains display markedly different neuropathogenic potential. The Brazilian strain (ZIKV^BR^), which emerged during the 2015–2016 outbreak, has been causally linked to CZS and microcephaly (Cugola et al. 2016), whereas the ancestral African strain (ZIKV^AF^) is associated with higher virulence and mortality but does not produce the same neurodevelopmental phenotype (Aubry et al. 2021; Tripathi et al. 2017). Identifying molecular signatures that distinguish these strain-specific effects, and that separate ZIKV neuropathogenesis from that of related flaviviruses such as DENV, is therefore of critical importance for the development of early diagnostic biomarkers and targeted neuroprotective strategies.

In this context, we recently identified Ndel1 (NudE neurodevelopment protein 1-like 1) oligopeptidase as a functional biomarker associated with CZS, showing predictive value for microcephaly and responsiveness to interferon treatment (Christoff et al. 2023). Ndel1 is a critical regulator of cytoskeletal dynamics in the developing brain, participating in microtubule organization, neuronal migration, and cortical progenitor cell function through its interaction with dynein and LIS1 (Shu et al. 2004; Hayashi et al. 2010). Loss of Ndel1 function disrupts neuronal positioning and cortical lamination, and pathogenic variants have been associated with cortical malformations in humans (Tsai et al. 2024), establishing a direct link between Ndel1 dysregulation and neurodevelopmental disease. We previously showed that Ndel1 oligopeptidase activity is reduced in embryos and neonatal mouse brains infected with ZIKV^BR^, correlating linearly with reduced brain size across multiple developmental stages (Christoff et al. 2023), supporting its potential use as a functional marker of microcephaly severity.

Here, we sought to determine whether Ndel1 modulation represents a conserved response to flavivirus infection or a virus- and strain-specific phenomenon. To this end, we compared the effects of ZIKV^BR^, ZIKV^AF^, and DENV on Ndel1 enzymatic activity and mRNA expression in neonatal mouse brains, and characterized the accompanying neuroinflammatory and interferon signaling responses. Elucidating these molecular mechanisms may contribute to the identification of early diagnostic biomarkers and support the development of targeted antiviral and neuroprotective strategies against these globally relevant pathogens.

## Material and Methods

### Animals

All animal procedures were approved by the Ethics Committees of the Federal University of Rio de Janeiro (UFRJ, protocol #A06/22-153-19) and the Federal University of São Paulo (UNIFESP, protocol #3,322,141,024), and were conducted in accordance with the Brazilian National Law (11.794/2008), the guidelines of the Brazilian National Council of Animal Experimentation (CONCEA), and the ARRIVE guidelines. Newborn (P0) Swiss mice of both sexes were used in this study, obtained from the UFRJ institutional animal facility (Centro de Ciências da Saúde, CCS). A total of 69 neonatal mice were used across three independent experiments: (1) ICV infection with ZIKV^BR^ or DENV (Mock n = 7, ZIKV^BR^ n = 7, DENV n = 7); (2) IP infection with ZIKV^BR^ or DENV (Mock n = 8, ZIKV^BR^ n = 11, DENV n = 10); (3) ICV infection with ZIKV^BR^ or ZIKV^AF^ for enzymatic activity, gene expression, and neuroinflammatory profiling (Mock n = 6, ZIKV^BR^ n = 6, ZIKV^AF^ n = 7). The mice were housed in standard cages (21 × 32 × 20 cm) with a maximum of four animals per cage, maintained under a 12-h light/dark cycle with ad libitum access to food and water. Animal well-being was monitored daily using a numerical scoring system based on body weight, general appearance, posture, activity, and breathing patterns. Although allocation of animals to treatment groups was not randomized, experimenters were blinded to sample identities during all enzymatic activity and gene expression analyses. At the end of each experiment, animals were anaesthetized with ketamine (120 mg/kg) and xylazine (12 mg/kg) prior to euthanasia by decapitation.

### ZIKV Propagation and Titration

Two Zika virus (ZIKV) strains were used in this study: a Brazilian isolate (ZIKV^BR^, strain ZIKV PE/243, GenBank accession number KX197192.1), and an African isolate (ZIKV^AF^, strain MR766, GenBank accession number NC_012532.1); Dengue virus (DENV) (DENV2 GenBank accession number 16681). The viruses were propagated in C6/36 mosquito cells at a multiplicity of infection (MOI) of 0.1. After 1 h of adsorption at 28 °C, the cultures were replenished with a medium containing 2% foetal bovine serum (FBS) and maintained for 5 days. The resulting supernatant was then centrifuged at 300 × g, filtered to remove debris and stored at -80 °C. Viral titres were determined by plaque assay in Vero cells, following standard procedures (Garcez et al. 2016; Contreras and Arumugaswami 2016) and our previous protocols (Christoff et al. 2023; Oyadomari et al. 2024). For mock infections, conditioned medium from uninfected C6/36 cells, prepared under identical conditions, was used as a negative control.

### ZIKV Infection

At P0, the pups were anaesthetised using hypothermia and injected intracerebroventricularly (ICV) with 30 PFU of either ZIKV^BR^, ZIKV^AF^ or a mock solution (1.5 μL). The brains were collected at either postnatal day 3 (P3) or 7 (P7), frozen and stored at – 80 °C. In some experiments, mice received intraperitoneal (IP) injections of 100 PFU of ZIKV^BR^ or the mock solution and tissues were harvested at P7.

### Preparation of Brain Homogenates

The brain tissues of mice were processed individually. The samples were homogenized on ice in a buffer solution of 50 mM Tris-HCl and 500 mM NaCl at pH 7.5 (4 mL of buffer solution per gram of tissue) using a Polytron homogenizer (Thermo Fisher Scientific, Waltham, MA, USA). The homogenates were subjected to three 30 s cycles with one-minute intervals. Following centrifugation at 20,000 × g for 10 min, at 4 °C, the pellet was resuspended in the same buffer and centrifuged again at 20,000 × g for 90 min, at 4 °C. The resulting pellet was resuspended in a buffer containing 0.5% Triton X-100, incubated on ice for one hour and then centrifuged at 5,000 × g for 10 min, at 4 °C. The resulting supernatant was collected for the determination of enzyme activity and protein concentration, quantified using the Bradford assay (Bio-Rad, Hercules, CA, USA).

### Reverse Transcription and Quantitative Real-Time PCR (RT-qPCR)

Total RNA was extracted from mouse brain tissue using Trizol™ Reagent (Thermo Fisher Scientific; Cat. No. 15596026) according to the manufacturer’s instructions. Tissue homogenization was performed with a Polytron® homogenizer to ensure efficient cell lysis and RNA release. RNA concentration and purity were assessed using a NanoDrop 2000c spectrophotometer (Thermo Fisher Scientific), with only samples exhibiting A260/280 and A260/230 ratios above 1.8 being selected for downstream analysis, a criterion that indicates minimal protein and phenol contamination. Complementary DNA (cDNA) was synthesized from 1000 ng of total RNA using the SuperScript™ IV First-Strand Synthesis System (Invitrogen, Cat. No. 4368814), following the manufacturer’s protocol. Quantitative PCR (qPCR) was carried out with Power SYBR™ Green PCR Master Mix (Applied Biosystems, Cat. No. 18091200) on a 7500 Real-Time PCR System (Applied Biosystems). Gene-specific primers (5’ → 3’ sequence) (Table 1) for the target mouse genes were purchased from Exxtend (São Paulo, Brazil).

**Table 1:** Mouse (Ms) targets

Gene	Forward	Reverse
*Tnf*	CCCTCACACTCAGATCATCTTCT	GCTACGACGTGGGCTACAG
*Il6*	TTCTTGGGACTGATGCTGGTG	CAGAATTGCCATTGCACAACTC
*Il1b*	GTAATGAAAGACGGCACACC	ATTAGAAACAGTCCAGCCCA
*Ifnar1*	CTGGTCTGTGAGCTGTACTT	TCCCCGCAGTATTGATGAGT
*Ifnar2*	CTATCGTAATGCTGAAACGG	GTAATTCCACAGTCTCTTCT
*Ifit1*	ACTGAGGCCCACATTTGAGA	GGGTGATTGCCTTGTCTTCTG
*Ndel1*	TGCACAGAGCTACAAGCAGGT	CTCGCTCCAGGTCATCATTG
*β-actin*	CAACTTGATGTATGAAGGCTTTGGT	ACTTTTATTGGTCTCAAGTCAGTGTACA

All reactions were performed in technical triplicates, with *β-actin* serving as the endogenous control. A melting curve analysis was conducted at the end of each run to confirm amplification specificity. The fluorescence threshold (Ct) was manually set based on appropriate negative and positive controls and was consistently applied across all samples and experimental conditions. Relative gene expression was calculated using the ^Δ^Ct and ^ΔΔ^Ct methods, with expression levels normalized to *β-actin* and expressed relative to the corresponding control group.

### Ndel1 Activity Measurements

Ndel1 oligopeptidase activity was determined using the fluorogenic FRET (Fluorescence Resonance Energy Transfer) substrate Abz-GFSPFRQ-EDDnp, as previously described (Nani et al. 2020). Reactions were performed in black 96-well plates in PBS (pH 7.4), containing 10 − 20 μg of total protein in a final volume of 200 μL. The increase in fluorescence, resulting from substrate cleavage by Ndel1 oligopeptidase, was monitored in an F-7000 fluorimeter (Hitachi Ltd., Tokyo, Japan) at 37 °C for 30 min, with readings taken every 60 s (λ_Ex_ 320 nm and λ_Em_ 420 nm). Quantification was performed using a standard curve generated with known concentrations of cleaved substrate. All samples were analysed in triplicate. Ndel1 activity was expressed as nM*min⁻^1^*mg⁻^1^ protein. Total protein concentration was determined using the Bradford method (Bio-Rad).

### Data and Statistical Analysis

Sample sizes were determined a priori using power analysis based on effect sizes estimated from pilot experiments and previously published data from our group (Christoff et al. 2023), targeting a power of 0.80 and a significance level of α = 0.05, following the guidelines described by Kadam and Bhalerao (2010). Sample sizes per experimental group were as follows: Ndel1 enzymatic activity, ICV experiment: n = 7 per group (Mock, ZIKV^BR^, DENV); IP experiment: Mock n = 8, ZIKV^BR^ n = 11, DENV n = 10; ZIKV^BR^ vs. ZIKV^AF^ activity: Mock n = 6, ZIKV^BR^ n = 6, ZIKV^AF^ n = 7; Ndel1 activity at P3 and P7: n = 5 per group; gene expression (Ndel1, cytokines, and interferon-pathway genes) in the ZIKV^BR^ vs. ZIKV^AF^ experiment: Mock n = 6, ZIKV^BR^ n = 6, ZIKV^AF^ n = 4. These sample sizes are also reported in each figure legend. All measurements were performed in technical triplicate; each data point in the graphs represents the mean of three technical replicates from one biological replicate (i.e., one individual animal). All data are expressed as mean ± SD and individual data points are shown in all graphs.

Animals were randomly assigned to experimental groups prior to infection. All enzymatic activity measurements and gene expression quantifications were performed under blinded conditions: biological samples were received and processed without knowledge of group identity, which was revealed only after all measurements and dosages were completed.

Prior to inferential testing, normality was assessed for each group independently using the Shapiro–Wilk test and the Kolmogorov–Smirnov test for groups with n < 8, and additionally with the D’Agostino–Pearson and Anderson–Darling tests for groups with n ≥ 8. Because Welch’s ANOVA and the Brown–Forsythe ANOVA test do not assume homogeneity of variance, no separate test for variance heterogeneity was required when these tests were applied; they were selected a priori as the primary tests for all multi-group comparisons to provide robustness against unequal variances. When one or more groups failed normality testing, the Kruskal–Wallis test was used instead. For two-group comparisons, the unpaired Welch’s t-test was applied, which likewise does not assume equal variances.

For experiments comparing three groups on Ndel1 enzymatic activity and gene expression, and on cytokine and interferon-pathway gene expression, Welch’s ANOVA and the Brown–Forsythe ANOVA test were applied in parallel. Post hoc comparisons against the mock control group were performed using Dunnett’s T3 test, which is appropriate for unequal variances. For *Il1b* expression in the ZIKV^BR^ vs. ZIKV^AF^ experiment, the Shapiro–Wilk test indicated non-normal distribution in the ZIKV^BR^ group (W = 0.713, p = 0.0085); accordingly, the Kruskal–Wallis test was applied, with Dunn’s test used for post hoc comparisons. *Ifnar2* expression in the same experiment was also analysed by Kruskal–Wallis with Dunn’s post hoc test. Two-group comparisons of Ndel1 activity at individual timepoints (P3 and P7) were performed using the unpaired Welch’s t-test.

All tests were two-tailed. The significance threshold was set at α = 0.05. Exact p-values are reported for all p > 0.001; values at or below 0.001 are reported as p < 0.001. Statistical analyses were performed using GraphPad Prism version 9.5.1 (GraphPad Software, San Diego, CA, USA).

## Results

### Ndel1 Enzymatic Activity in Neonatal Mouse Brains is Reduced by ZIKV, but not by DENV Infection

To determine whether modulation of Ndel1 oligopeptidase activity is specific to ZIKV infection or represents a broader response to flaviviral infection, neonatal Swiss mice (P0) were infected via intracerebroventricular (ICV) injection with the Brazilian ZIKV strain (ZIKV^BR^) or dengue virus (DENV). Mock control animals received equivalent injections of uninfected C6/36 conditioned medium. Brain tissues were collected at 7 days post-infection (dpi) for enzymatic activity analysis.

**Fig. 1:**
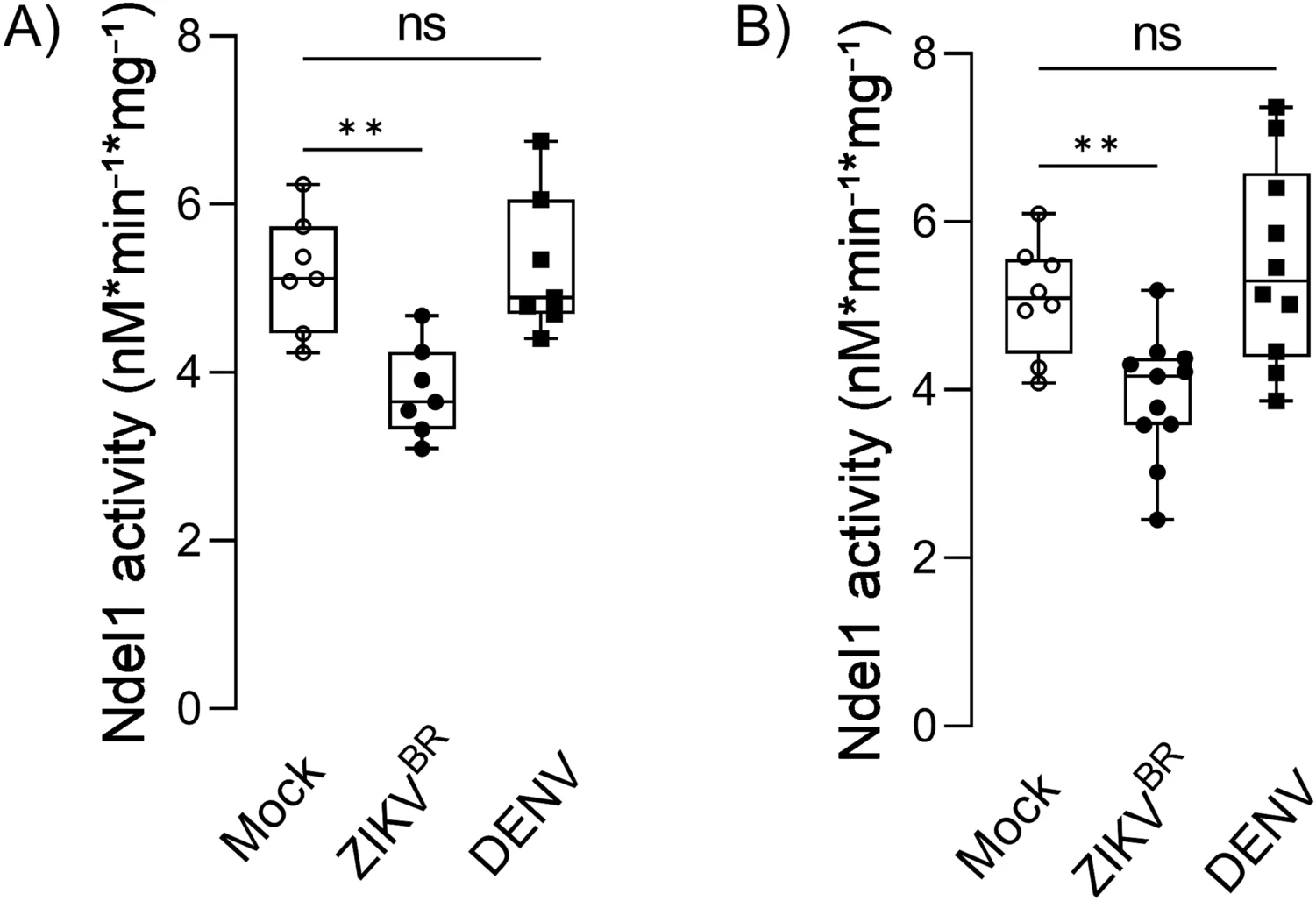
Ndel1 activity is reduced in neonatal mouse brains by ZIKV^BR^, but not by DENV infection. Ndel1 enzymatic activity was measured in brain homogenates collected at P7 from neonatal mice infected at P0 with ZIKV^BR^ or DENV via **A** intracerebroventricular (ICV) or **B** intraperitoneal (IP) routes and compared with mock-infected controls. For the ICV model, biological replicates consisted of individual animals (Mock, n = 7; ZIKV^BR^, n = 7; DENV, n = 7). For the IP model, biological replicates consisted of individual animals (Mock, n = 8; ZIKV^BR^, n = 11; DENV, n = 10). Ndel1 activity was determined from three technical replicates per animal; each data point represents the mean of these technical triplicates for a single biological replicate. Data are presented as mean ± SD with individual data points shown. Normality was assessed using the Shapiro–Wilk and Kolmogorov–Smirnov tests (ICV model) and additionally the D’Agostino–Pearson and Anderson–Darling tests (IP model); all groups passed normality testing. Statistical comparisons were performed using Welch’s ANOVA and the Brown–Forsythe ANOVA test, followed by Dunnett’s T3 post hoc test. ns, not significant; **p < 0.01

Consistent with our previous findings (Christoff et al. 2023), Welch’s ANOVA revealed a significant effect of infection on Ndel1 activity (W(2, 11.63) = 11.79, *p* = 0.0016). Post hoc comparisons using Dunnett’s T3 test demonstrated that Ndel1 activity was significantly reduced in ZIKV^BR^-infected animals (3.78 ± 0.54 nM*min⁻^1^*mg⁻^1^) compared with mock controls (5.18 ± 0.69 nM*min⁻^1^*mg⁻^1^; adjusted p = 0.0029), whereas no significant difference was observed between DENV-infected animals (5.28 ± 0.84 nM*min⁻^1^*mg⁻^1^) and mock controls (adjusted p = 0.9660) (Fig. 1A).

To evaluate whether this effect was dependent on the route of infection, neonatal mice were also infected via the intraperitoneal (IP) route. Welch’s ANOVA again revealed a significant effect of infection on Ndel1 activity (W(2, 16.51) = 8.97, p = 0.0023), with Dunnett’s T3 post hoc analysis confirming a significant reduction in ZIKV^BR^-infected animals (3.92 ± 0.76 nM*min⁻^1^*mg⁻^1^) relative to mock controls (5.08 ± 0.70 nM*min⁻^1^*mg⁻^1^; adjusted p = 0.0054), but no significant difference for DENV-infected animals (5.49 ± 1.16 nM*min⁻^1^*mg⁻^1^; adjusted p = 0.5971) (Fig. 1B).

Together, these findings demonstrate that Ndel1 enzymatic downregulation is selectively associated with ZIKV infection and occurs independently of the infection route, supporting its potential relevance as a functional marker of ZIKV-induced neuropathogenesis.

### Strain-Specific Effects of ZIKV on Ndel1 Activity and Expression

To further investigate whether Ndel1 modulation is strain-dependent and to strengthen its potential relevance as a biomarker associated with microcephaly, we compared the effects of the Brazilian (ZIKV^BR^) and African (ZIKV^AF^) ZIKV strains in neonatal mice. Due to the high mortality associated with ZIKV^AF^ infection, which is consistent with its reported higher virulence (Aubry et al. 2021), analyses at later time points (e.g., post-natal day 7—P7) were not feasible for this strain. Therefore, early time points were selected to allow strain comparison under comparable survival conditions. Notably, previous findings by Christoff et al. demonstrated that ZIKV^BR^ infection also induces a reduction in Ndel1 enzymatic activity at early postnatal stages (postnatal day 3—P3), supporting the evaluation of this time point.

**Fig. 2:**
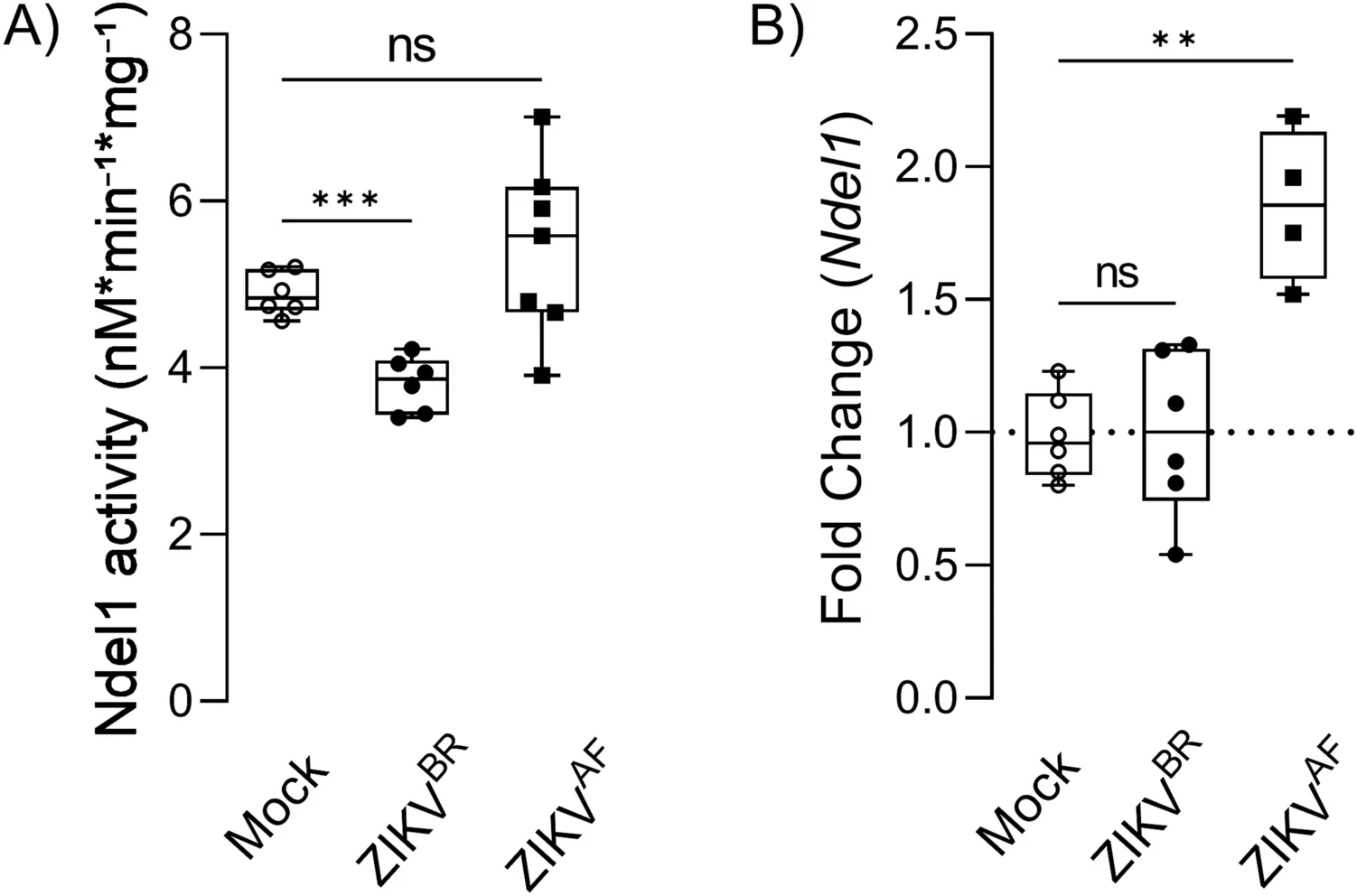
Strain-specific effects of ZIKV on activity and expression of Ndel1. Neonatal mice were infected at P0 via intracerebroventricular (ICV) injection with either the Brazilian ZIKV strain (ZIKV^BR^) or the African ZIKV strain (ZIKV^AF^). Brain tissue was collected at postnatal day 3 (P3, corresponding to 3 days post-infection). **A** Ndel1 enzymatic activity measured in brain homogenates from mock-infected (n = 6), ZIKV^BR^-infected (n = 6), and ZIKV^AF^-infected animals (n = 7). **B** Relative Ndel1 mRNA expression determined by RT-qPCR using the 2^−ΔΔCt^ method, normalized to the mock control group (Mock, n = 6; ZIKV^BR^, n = 6; ZIKV^AF^, n = 4). Each data point represents the mean of three technical replicates from a single biological replicate. Data are presented as mean ± SD with individual data points shown. Normality was assessed using the Shapiro–Wilk and Kolmogorov–Smirnov tests; all groups passed normality testing. Welch’s ANOVA and the Brown–Forsythe ANOVA test were applied a priori as primary tests for multi-group comparisons, given their robustness to variance heterogeneity, followed by Dunnett’s T3 post hoc test. ns, not significant; *p < 0.05; **p < 0.01; ***p < 0.001

At P3, Welch’s ANOVA revealed a significant effect of infection on Ndel1 activity (W(2, 10.00) = 21.48, p = 0.0002). Post hoc comparisons using Dunnett’s T3 test demonstrated that Ndel1 activity was significantly reduced in ZIKV^BR^-infected animals (3.81 ± 0.33 nM*min⁻^1^*mg⁻^1^) compared with mock controls (4.89 ± 0.25 nM*min⁻^1^*mg⁻^1^; adjusted p = 0.0003), whereas ZIKV^AF^-infected animals (5.44 ± 1.12 nM*min⁻^1^*mg⁻^1^) did not differ significantly from mock controls (adjusted p = 0.3881) (Fig. 2A).

To investigate whether this enzymatic reduction was accompanied by transcriptional changes, Ndel1 mRNA expression was assessed at the same timepoint. Welch’s ANOVA revealed a significant effect of infection on Ndel1 expression (W(2, 6.702) = 14.19, p = 0.0039). Post hoc analysis using Dunnett’s T3 test showed that expression was unchanged in ZIKV^BR^-infected animals (1.00 ± 0.32-fold) relative to mock controls (0.99 ± 0.17-fold; adjusted p = 0.9958), whereas ZIKV^AF^ infection significantly upregulated Ndel1 mRNA (1.86 ± 0.29-fold; adjusted p = 0.0098) (Fig. 2B).

The dissociation between enzymatic activity and mRNA expression in ZIKV^BR^-infected animals suggests that the reduction in Ndel1 activity is not driven by transcriptional downregulation, pointing instead to post-transcriptional or post-translational mechanisms. In contrast, ZIKV^AF^ infection did not alter Ndel1 enzymatic activity relative to mock controls. This lack of modulation supports the notion that the reduction in Ndel1 activity is a ZIKV^BR^-specific effect, rather than a general consequence of flavivirus infection.

### Ndel1 Oligopeptidase Activity, but not its mRNA Expression, Reflects Early ZIKV-Induced Molecular Alterations

We previously reported a strong correlation between reduced Ndel1 enzymatic activity and decreased brain size in animals infected at different developmental stages, supporting Ndel1 activity as a potential early biomarker for microcephaly (Christoff et al. 2023). Based on these findings, we next investigated whether Ndel1 mRNA expression could also serve as a biomarker in this context.

At P3, Ndel1 enzymatic activity was already significantly reduced in ZIKV^BR^-infected animals compared with mock controls (3.81 ± 0.33 vs. 4.89 ± 0.25 nM*min⁻^1^*mg⁻^1^, respectively; Dunnett’s T3 adjusted p = 0.0003) (Fig. 2A). In contrast, Ndel1 mRNA expression was not altered at this time point (1.00 ± 0.32 vs. 0.99 ± 0.17 fold-change, respectively; Dunnett’s T3 adjusted p = 0.9958) (Fig. 2B).

**Fig. 3:**
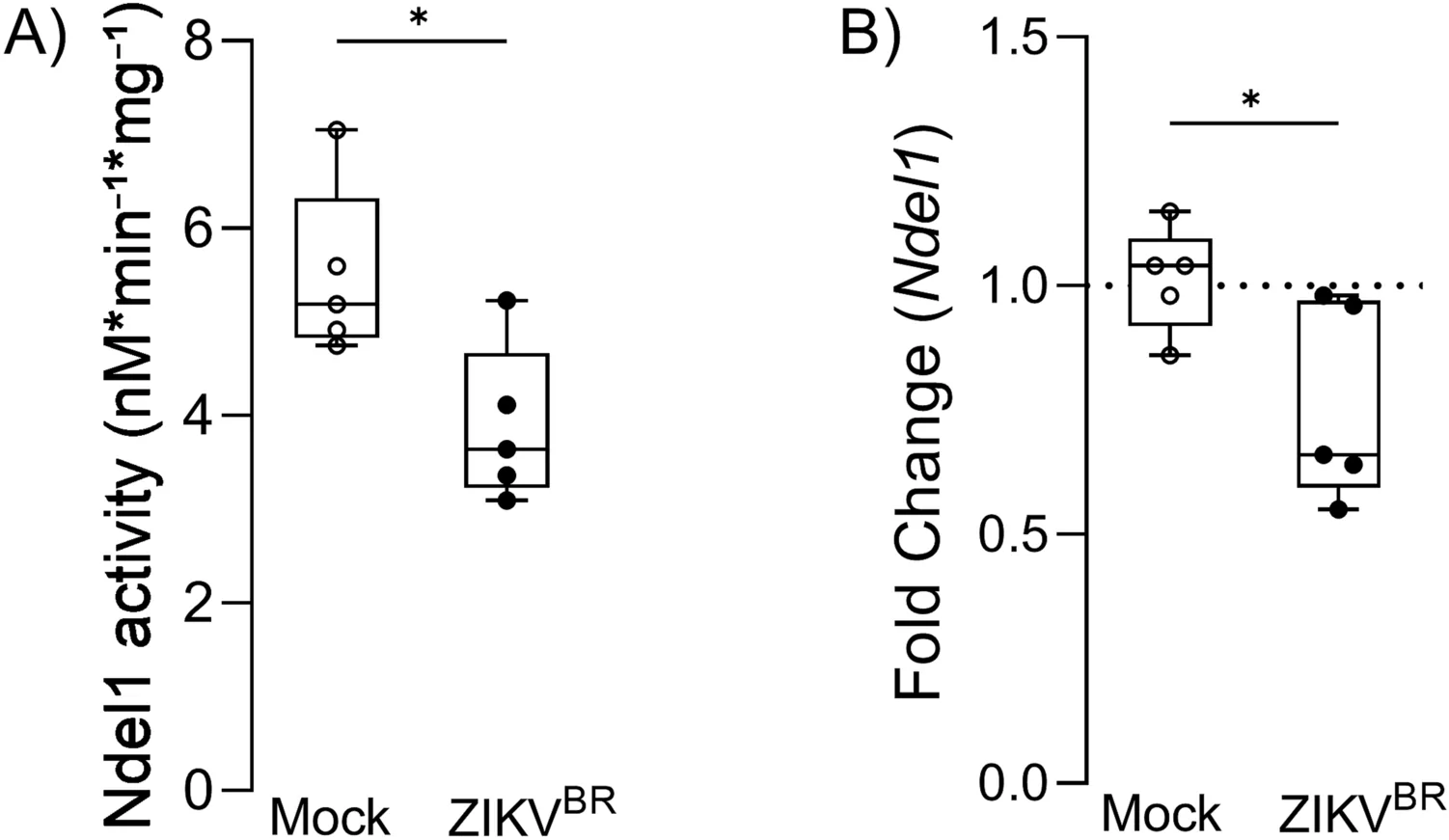
Ndel1 activity serves better as an early marker than its mRNA expression. Neonatal mice were infected at P0 via intracerebroventricular (ICV) injection with the Brazilian ZIKV strain (ZIKV^BR^). Brain tissue was collected for analysis at 7 days post-infection (dpi). **A** Ndel1 activity measured in brain homogenates from mock (n = 5), ZIKV^BR^-infected (n = 5). **B** Relative *Ndel1* mRNA expression determined by RT-qPCR and calculated using the 2^−ΔΔCt^ method, normalized to the corresponding mock control group (mock, n = 5; ZIKV^BR^, n = 5). Each data point represents the mean of three technical replicates from a single biological replicate. Data are presented as mean ± SD with individual data points shown. Normality was assessed using the Shapiro–Wilk and Kolmogorov–Smirnov tests; all groups passed normality testing. Statistical comparisons were performed using a two-tailed unpaired Welch’s t-test. ns, not significant; *p < 0.05

Analysis of Ndel1 mRNA expression and oligopeptidase activity revealed a concordant reduction only at P7. At this time point, both Ndel1 activity (4.15 ± 0.99 vs. 5.48 ± 0.83 nM*min⁻^1^*mg⁻^1^; Welch’s t-test, t(9.70) = 2.53, p = 0.0303) (Fig. 3A) and Ndel1 mRNA expression (0.76 ± 0.20 vs. 1.01 ± 0.10 fold-change; Welch’s t-test, t(6.11) = 2.55, p = 0.0428) (Fig. 3B) were significantly reduced in ZIKV-infected animals compared with mock controls.

Together, these results indicate that functional enzymatic activity changes precede transcriptional alterations, supporting Ndel1 activity as a more sensitive early indicator of ZIKV^BR^-associated neurodevelopmental alterations.

### Ndel1 Downregulation Occurs Independently of Neuroinflammatory Responses

Given the distinct patterns observed for Ndel1 enzymatic activity and mRNA expression following ZIKV^BR^ and ZIKV^AF^ infection, and considering the well-established stronger innate immune activation induced by ZIKV^AF^ compared with ZIKV^BR^, we next evaluated the expression of classical inflammatory biomarkers in neonatal brains at P3 following infection with these ZIKV strains.

**Fig. 4:**
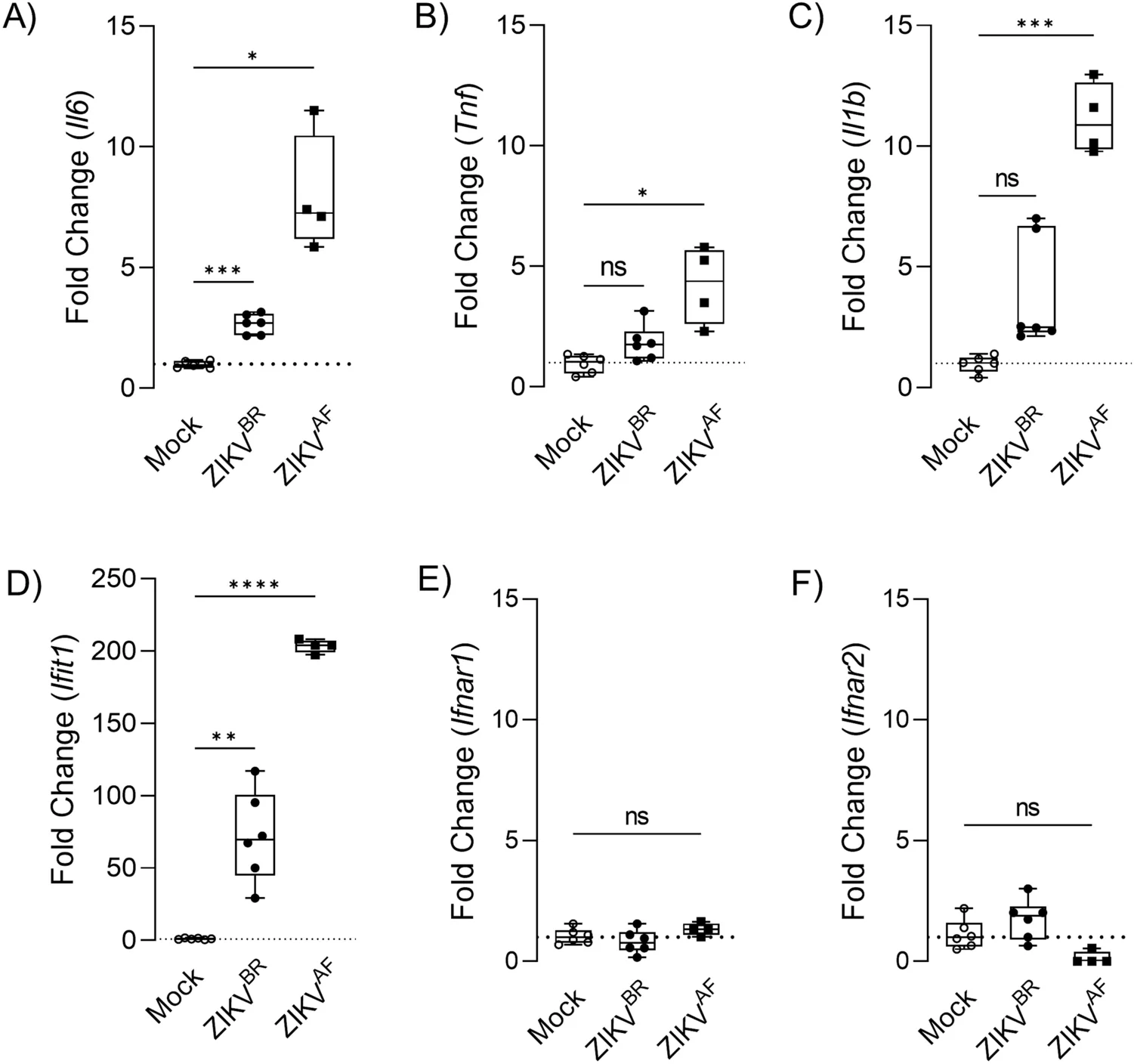
Strain-specific effects of ZIKV on modulation of pro-inflammatory cytokines. Neonatal mice were infected at P0 via ICV injection with either the Brazilian ZIKV strain (ZIKV^BR^) or the African ZIKV strain (ZIKV^AF^). Brain tissue was collected at postnatal day 3 (P3, corresponding to 3 days post-infection) for gene expression analysis. Fold-change values were calculated using the 2^−ΔΔCt^ method and normalized to the mock control group. Expression levels of **A** *Il6*, **B** *Tnf*, **C** *Il1b*, **D** *Ifit1*, **E** *Ifnar1*, and **F** *Ifnar2* are shown. Biological replicates per group: Mock, n = 6; ZIKV^BR^, n = 6; ZIKV^AF^, n = 4. Each data point represents the mean of three technical replicates from a single biological replicate. Data are presented as mean ± SD with individual data points shown. Normality was assessed for each group using the Shapiro–Wilk and Kolmogorov–Smirnov tests. For *Il6*, *Tnf*, *Ifit1*, and *Ifnar1*, all groups passed normality testing; statistical comparisons were performed using Welch’s ANOVA and the Brown–Forsythe ANOVA test, followed by Dunnett’s T3 post hoc test. For *Il1b*, the ZIKV^BR^ group failed normality testing (Shapiro–Wilk: W = 0.713, p = 0.0085); the Kruskal–Wallis test followed by Dunn’s multiple-comparisons test was therefore applied. *Ifnar2* was also analysed using the Kruskal–Wallis test with Dunn’s post hoc test, given the small sample size in the ZIKV^AF^ group (n = 4). ns, not significant; *p < 0.05; **p < 0.01; ***p < 0.001; ****p < 0.0001.

ZIKV^BR^ infection induced a significant increase in *Il6* expression (2.67 ± 0.43-fold) compared with mock controls (0.98 ± 0.14-fold; Dunnett’s T3 adjusted p = 0.0002), whereas *Tnf* expression (1.83 ± 0.72-fold vs. 0.95 ± 0.38-fold in mock; adjusted p = 0.0683) and *Il1b* expression did not differ significantly from mock controls (Dunn’s adjusted p = 0.0581) (Fig. 4A–C). In contrast, ZIKV^AF^ infection induced a marked inflammatory response, with significant increases in *Il6* (7.97 ± 2.43-fold; adjusted p = 0.0187), *Tnf* (4.22 ± 1.61-fold; adjusted p = 0.0492), and *Il1b* expression (11.12 ± 1.49-fold; Dunn’s adjusted p = 0.0007) compared with mock controls (Fig. 4A–C).

A robust induction of the interferon-stimulated gene *Ifit1* was observed in both infected groups. Welch’s ANOVA revealed a significant effect of infection (W(2, 5.039) = 3531, p < 0.001), with Dunnett’s T3 post hoc comparisons confirming significant upregulation in both ZIKV^BR^-infected animals (71.87 ± 32.17-fold; adjusted p = 0.0049) and ZIKV^AF^-infected animals (203.50 ± 4.50-fold; adjusted p < 0.001) relative to mock controls (1.08 ± 0.47-fold) (Fig. 4D).

No significant differences were detected among groups in *Ifnar1* expression (Welch’s ANOVA, W(2, 8.319) = 2.539, p = 0.1378) (Fig. 4E). Although the Kruskal–Wallis test indicated an overall significant difference in *Ifnar2* expression (H(2) = 8.619, p = 0.0055), post hoc Dunn’s comparisons revealed no significant differences between mock and either ZIKV^BR^ (adjusted p = 0.7255) or ZIKV^AF^ (adjusted p = 0.0733) (Fig. 4F).

Altogether, these findings indicate that the decrease in Ndel1 activity observed following ZIKV^BR^ infection occurs concurrently with, rather than as a downstream consequence of, the neuroinflammatory response, since both are already detectable at P3. Both ZIKV^BR^ and ZIKV^AF^ triggered robust cytokine responses, however, only ZIKV^BR^ infection resulted in reduced Ndel1 activity. This dissociation supports the notion that Ndel1 modulation is a virus strain-specific effect rather than a secondary consequence of the inflammatory environment.

## Discussion

In a previous study, we demonstrated a strong correlation between Ndel1 oligopeptidase activity and brain size in animals infected with ZIKV^BR^ across multiple developmental stages, independent of viral load and route of infection (Christoff et al. 2023), supporting this enzymatic readout as an early biomarker of microcephaly, a hallmark neurological outcome specifically associated with ZIKV^BR^ infection during the Brazilian outbreak. Building on these findings, the present study sought to determine whether Ndel1 modulation represents a conserved feature of flaviviral infection or a virus- and strain-specific phenomenon. Our data demonstrate that Ndel1 downregulation is not a general consequence of flavivirus infection, but rather a selective feature restricted to the Brazilian ZIKV strain, as neither the African strain (ZIKV^AF^) nor Dengue virus (DENV) altered Ndel1 oligopeptidase activity under the same experimental conditions.

Ndel1 activity was significantly reduced in ZIKV^BR^-infected animals compared with mock controls regardless of the route of infection, with consistent reductions observed following both intracerebroventricular and intraperitoneal delivery, while DENV-infected animals did not differ from mock controls in either model. This route-independence reinforces the specificity of the effect and its dissociation from local viral delivery. Similarly, in the strain comparison experiment, ZIKV^BR^ reduced Ndel1 activity at P3 whereas ZIKV^AF^ infection had no significant effect, despite being associated with higher virulence and mortality in neonatal mice (Aubry et al. 2021).

At the transcriptional level, *Ndel1* mRNA expression remained unaltered in ZIKV^BR^-infected animals relative to mock controls at P3, despite the concurrent reduction in enzymatic activity. Conversely, ZIKV^AF^ induced a significant increase in *Ndel1* mRNA expression at P3 without any corresponding change in enzymatic activity. Thus, under both infection conditions, transcript abundance failed to predict enzymatic activity. This double dissociation indicates that the regulation relevant to Ndel1 catalytic function at this stage is not transcriptional, and points instead to post-transcriptional control, either of Ndel1 protein abundance or of the catalytic activity of the existing protein pool. Regarding the latter possibility, a substantial proportion of Ndel1 protein exists in complex with cytosolic partners such as DISC1, which negatively regulates its oligopeptidase activity (Hayashi et al. 2005); dissociations between *Ndel1* transcript levels and enzymatic activity have also been reported previously in neuropsychiatric contexts (Ota et al. 2015; Gouvea et al. 2016; Dal Mas et al. 2019). By P7, *Ndel1* mRNA expression was also significantly reduced in ZIKV^BR^-infected animals, paralleling the decrease in enzymatic activity; given that the activity phenotype is already established at P3 in the absence of any transcript change, this later transcriptional decline is more plausibly a downstream consequence of infection progression than the primary driver of the enzymatic effect. Together, these findings reinforce the concept that Ndel1 enzymatic activity provides a more sensitive and functionally relevant readout than mRNA levels in the context of neuronal regulation.

The strain-specific reduction in Ndel1 activity directly parallels the neurodevelopmental phenotype associated with ZIKV^BR^, as this lineage has been causally linked to the emergence of congenital microcephaly (Cugola et al. 2016). In this context, our findings reinforce that Ndel1 downregulation is not only a molecular signature of ZIKV^BR^ infection, but also a functional correlate of the neurodevelopmental impairment uniquely associated with this strain. The absence of Ndel1 modulation in ZIKV^AF^-infected animals, despite robust viral replication and higher associated mortality, underscores that enzymatic downregulation is not a passive consequence of viral pathogenicity, but rather a strain-specific host-virus interaction.

The strain-specific reduction in Ndel1 activity may be mechanistically relevant to the unique capacity of ZIKV^BR^ to induce congenital microcephaly (Cugola et al. 2016; Cauchemez et al. 2016). Ndel1 is a critical regulator of cytoskeletal dynamics in the developing brain, promoting neurite outgrowth, neuronal migration, and cortical progenitor integrity through its role in microtubule organization and dynein-mediated transport (Shu et al. 2004; Chansard et al. 2011). The absence of Ndel1 modulation in ZIKV^AF^-infected animals, which do not recapitulate the microcephalic phenotype associated with ZIKV^BR^ (Aubry et al. 2021, Li et al. 2017), further supports this interpretation. Whether this enzymatic reduction is a direct mediator or an early correlate of ZIKV^BR^-specific cortical pathology remains to be established. Future studies should investigate whether ZIKV^BR^-specific viral factors interact with Ndel1 regulatory partners or interfere with post-translational modification pathways known to govern its enzymatic function.

Consistent with the established role of innate immunity in flavivirus infections, we observed induction of *Il6* expression in ZIKV^BR^-infected brains at P3 relative to mock controls. This is consistent with prior comparisons of transcript and protein-level cytokine induction in ZIKV-infected mouse brain and other neurotropic flavivirus models, in which mRNA changes closely paralleled protein output (Jeong et al. 2023; Tripathi et al. 2017; Li et al. 2004). However, this inflammatory profile did not parallel the modulation of Ndel1 activity, indicating that Ndel1 downregulation is not a direct consequence of pro-inflammatory signaling. This dissociation is particularly evident in ZIKV^AF^-infected animals, which displayed a robust inflammatory response at P3, with significant increases in *Il6, Tnf,* and *Il1b*, without any corresponding change in Ndel1 activity, further reinforcing the strain-specific nature of this enzymatic modulation.

In parallel, we observed robust upregulation of the interferon-stimulated gene *Ifit1* in response to both strains at P3, with ZIKV^AF^ eliciting a substantially stronger induction than ZIKV^BR^. Notably, *Ifnar1* and *Ifnar2* expression was not significantly upregulated at this early timepoint, suggesting that ISG induction may occur through receptor-independent or non-canonical mechanisms in this model, as has been described for related flaviviruses (Kumar et al. 2016).

Importantly, the profile of Ndel1 modulation did not coincide with the inflammatory or interferon-stimulated gene responses observed at P3, indicating that Ndel1 downregulation occurs independently of both canonical and non-canonical interferon signaling pathways at this stage. By P7, *Ndel1* mRNA expression was also significantly reduced, paralleling the decrease in enzymatic activity, suggesting that transcriptional regulation of Ndel1 becomes engaged as infection progresses. Taken together, these data suggest a functionally uncoupled host response, in which early Ndel1 downregulation at P3 occurs concurrently with, rather than as a direct consequence of, cytokine induction and interferon signaling, pointing toward a direct or indirect interaction between ZIKV^BR^-specific molecular determinants and the regulatory machinery governing Ndel1 function.

Several limitations of the present study warrant consideration. First, while our data localize Ndel1 regulation to the post-transcriptional level and dissociate it from inflammatory and interferon signaling, the upstream molecular events linking ZIKV^BR^-specific viral factors to Ndel1 enzymatic modulation remain to be identified. In particular, our data do not distinguish between a reduction in Ndel1 protein abundance and preserved abundance with reduced catalytic function, for example through sequestration by cytosolic partners such as DISC1 or through post-translational modification. Future studies quantifying Ndel1 protein abundance and its association with regulatory partners will be required to resolve this. Finally, all experiments were conducted in a single neonatal mouse model, and the translational relevance of these findings to human congenital Zika syndrome will require validation in additional model systems, including human brain organoids and non-human primate models.

Our results demonstrate that Ndel1 downregulation is not a conserved feature of flavivirus infection, nor a universal response to ZIKV, but rather a strain-specific signature of ZIKV^BR^-induced neuropathogenesis. While both ZIKV strains elicited robust inflammatory and interferon responses, only ZIKV^BR^ infection led to a consistent reduction in Ndel1 enzymatic activity, an effect that was independent of route of infection, dissociated from transcriptional regulation, and not recapitulated by DENV or ZIKV^AF^ under identical experimental conditions. Importantly, this enzymatic reduction aligns with the unique capacity of ZIKV^BR^ to induce congenital microcephaly (Cugola et al. 2016; Cauchemez et al. 2016), reinforcing Ndel1 activity as a functional correlate of this neurodevelopmental outcome. These findings establish Ndel1 enzymatic activity as a strain-specific and early marker of ZIKV^BR^-induced neuropathogenesis, providing mechanistic insight into the molecular basis of microcephaly associated with the Brazilian outbreak.

## Data Availability

All data generated or analysed during this study are included in this published article.

## Abbreviations

BBB: Blood–brain barrier
C6/36: Aedes albopictus mosquito cell line
CNS: Central nervous system
Ct: Cycle threshold
CZS: Congenital Zika syndrome
DENV: Dengue virus
DNA: Deoxyribonucleic acid
dpi: Days post-infection
EBOV: Ebola virus
FBS: Foetal bovine serum
FRET: Fluorescence resonance energy transfer
ICV: Intracerebroventricular
IFIT1: Interferon-induced protein with tetratricopeptide repeats 1
IFN-β: Interferon beta
IFN-I: Type I interferon
IFNAR1: Interferon alpha/beta receptor subunit 1
IFNAR2: Interferon alpha/beta receptor subunit 2
IL-1β: Interleukin-1 beta
IL-6: Interleukin-6
IP: Intraperitoneal
ISG: Interferon-stimulated gene
ISGs: Interferon-stimulated genes
JAK-STAT: Janus kinase-signal transducer and activator of transcription pathway
MERS: Middle east respiratory syndrome
MOI: Multiplicity of infection
Ndel1: NudE neurodevelopment protein 1-like 1
P0: Postnatal day 0
P3: Postnatal day 3
P7: Postnatal day 7
PFU: Plaque-forming unit
qPCR: Quantitative polymerase chain reaction
RT-qPCR: Reverse transcription quantitative PCR
SARS: Severe acute respiratory syndrome
SARS-CoV-2: Severe Acute Respiratory Syndrome Coronavirus 2
STAT: Signal transducer and activator of transcription
TNF-α: Tumor necrosis factor-alpha
WHO: World Health Organization
ZIKV: Zika virus
ZIKV^AF^: African strain of Zika virus
ZIKV^BR^: Brazilian strain of Zika virus
nM*min⁻^1^*mg⁻^1^: Nanomolar per minute per milligram of protein
